# Universal barcoding regions, *rbc*L, *mat*K and *trn*H*-psb*A do not discriminate *Cinnamomum* species in Sri Lanka

**DOI:** 10.1371/journal.pone.0245592

**Published:** 2021-02-10

**Authors:** C. H. W. M. R. Bhagya Chandrasekara, D. Nathasha U. Naranpanawa, B. Supun Bandusekara, D. K. N. G. Pushpakumara, D. Siril A. Wijesundera, Pradeepa C. G. Bandaranayake

**Affiliations:** 1 Agricultural Biotechnology Centre, Faculty of Agriculture, University of Peradeniya, Peradeniya, Sri Lanka; 2 Postgraduate Institute of Science, University of Peradeniya, Peradeniya, Sri Lanka; 3 Postgraduate Institute of Agriculture, University of Peradeniya, Peradeniya, Sri Lanka; 4 Department of Crop Science, Faculty of Agriculture, University of Peradeniya, Peradeniya, Sri Lanka; 5 National Institute of Fundamental Studies, Kandy, Sri Lanka; Institute for Biological Research, SERBIA

## Abstract

The genus *Cinnamomum* consists of about 250 species spread globally. Out of these, *C*. *verum* (*C*. *zeylanicum*), also known as true cinnamon or Ceylon cinnamon, has gained worldwide attention due to its culinary uses and medicinal values. Sri Lanka is the largest true cinnamon producer in the world and accounts for about 80–90% of global production. Other than the cultivated species, Sri Lankan natural vegetation is home to seven endemic wild species of the genus *Cinnamomum*. While these are underutilized, proper identification and characterization are essential steps in any sustainable conservation and utilization strategies. Currently, species identification is purely based on morphological traits, and intraspecific diversity has made it more challenging. In this study, all the eight *Cinnamomum* species found in Sri Lanka, *C*. *capparu-coronde*, *C*. *citriodorum C*. *dubium*, *C*. *litseifolium*, *C*. *ovalifolium*, *C*. *rivulorum*, *C*. *sinharajaense*, and *C*. *verum* were collected in triplicates and identified using typical morphological traits. DNA extracted with the same collection was assessed with universal barcoding regions, *rbc*L, *mat*K, and *trn*H*-psb*A. While no intraspecific sequence differences were observed in *C*. *citriodorum*, *C*. *rivulorum*, and *C*. *verum*, the others had polymorphic sites in one, two, or all regions assessed. Interestingly, two individuals of *C*. *sinharajaense* had identical barcodes to the cultivated species *C*. *verum*, while the other one had one variable cite in *mat*K region and three cites in *trn*H*-psb*A reigon. Further, one *C*. *dubium* and one *C*. *capparu-coronde* accession each had identical, *rbc*L, and *trn*H*-psb*A sequences while those had only a single nucleotide variation observed in *mat*K region. Overall, the phylogeny of *Cinnamomum* species found in Sri Lanka could not be completely resolved with DNA barcoding regions studied.

## Introduction

The genus *Cinnamomum* consists of approximately 250 species [1]. Originally, the genus *Cinnamomum* was considered a purely Asiatic genus recorded only in the eastern hemisphere, specifically in the Asia Pacific region [2]. Later, several South American species previously included in *Phoebe* were transferred to *Cinnamomum* [3]. However, it was shown that these New World species are closer to the Neotropical genus *Aiouea* than to the Asian species [4]. Nevertheless, some *Cinnamomum* species have been used for various purposes since ancient times. For example, cinnamon was used to preserve meat and to retard the growth of bacteria. In ancient Rome, it had been used medicinally for cold and flu, as well as to treat diseases associated with the digestive system [5].

*Cinnamomum verum* J. Presl and *C*. *aromaticum* Nees are traded in local and world markets as cinnamon of commerce [6]. Of these, *C*. *verum*, also known as Ceylon cinnamon, is considered as the true cinnamon. The species is also known by its synonym *C*. *zeylanicum*, which was published only a few months later than *C*. *verum*. In addition to being used as a spice and a confectionary flavoring agent, *C*. *verum* has been used as an anti-inflammatory, anti-termitic, nematicidal, anti-mosquito, larvicidal, insecticidal, anti-mitotic, and anti-cancer agent. Cinnamon has also been traditionally used as tooth powder and to treat toothaches [7–12].

*Cinnamomum verum* is endemic to Sri Lanka and is originally found in upcountry rainforests. Portuguese traders brought it under cultivation and later planted it in the coastal areas. *Cinnamomum verum* was introduced to India in the 19^th^ century by taking seeds from Sri Lanka and planting them in the Western Ghats in Kerala and Tamil Nadu states [2]. Nevertheless, Sri Lanka is the largest true cinnamon producer in the world, accounting for 80–90% of global cinnamon production, earning US$ 190 million foreign exchange by exporting 17,500 metric tons in 2018. This is a 13% increment when compared to exports made in 2017, as of the Department of Export Agriculture, Sri Lanka.

Other than *C*. *verum*, there are seven endemic wild species of *Cinnamomum* in Sri Lanka. They are, *C*. *capparu-coronde* Blume, *C*. *citriodorum* Thwaites, *C*. *dubium* Nees, *C*. *litseifolium* Thwaites, *C*. *ovalifolium* Wight, *C*. *rivulorum* Kosterm. and *C*. *sinharajaense* Kosterm [13, 14]. Some of them are restricted to unique environmental conditions whereas others are distributed in a wide range of environmental conditions. For example, *C*. *ovalifolium* is only found in upcountry rainforests such as Horton plains and Sri Pada mountain, whereas *C*. *litseifolium* is widely found in Kandy, Badulla, Rathnapura, and Nuwara Eliya districts [15, 16]. While some of these species are utilized in Ayurvedic and traditional medicine [17], others are not in use so far. Species such as *C*. *citriodorum*, *C*. *dubium*, *C*. *litseifolium*, and *C*. *rivulorum* have unique chemical profiles while species such as *C*. *sinharajaense*, *C*. *capparu-coronde* have similar chemical constituents to *C*. *verum* [18, 19].

Currently accepted species classification and identification of *Cinnamomum* are purely based on morphological traits such as leaf arrangement, flush color, leaf shape, apex and venation [20, 21]. While *C*. *litseifolium*, *C*. *citriodorum*, *C*. *sinharajaense*, and *C*. *capparu-coronde* are easily distinguishable using leaf morphological traits, identification of others, such as *C*. *rivulorum* and *C*. *dubium* are not that straightforward [22]. Intraspecific diversity of morphological traits makes the identification even more difficult [22].

DNA barcoding, on the other hand, is a universally accepted methodology for molecular level identification of species [23–25]. Most botanists have used the chloroplast coding regions *rbc*L and *mat*K together with the *trn*H-*psb*A intergenic region as in DNA barcoding [26]. Molecular identification has also been implemented to assess both intra and interspecific genetic diversity of *Cinnamomum* to a certain extent. For example, Abeysinghe and colleagues utilized the chloroplast regions, the *trnL* intron, the *trn*T*-trn*L, *trn*L*-trn*F, and *trn*H*-psb*A intergenic spacers, as well as the internal transcribed spacer (ITS) of nuclear ribosomal DNA (rDNA) for identification of several wild *Cinnamomum* species, *C*. *citriodorum*, *C*. *capparu-coronde*, *C*. *dubium*, *C*. *litseifolium*, *C*. *rivulorum*, *C*. *sinharajaense*, *C*. *ovalifolium*, an unknown *Cinnamomum* species, and *C*. *verum* [27]. Accordingly, the chloroplast regions alone could not resolve some of the phylogenetic relationships among these species. The sequences of the ITS region turned out to be more useful for the identification of species. Since there was low variation among chloroplast regions studied, Abeysinghe and colleagues have used random amplified polymorphic DNA (RAPD) and sequence-related amplified polymorphic markers (SRAP) to study genetic diversity among selected species in their next study [28]. However, no follow-up studies were done including all the species. In elsewhere, *Cinnamomum* species used in traditional medicine have been assessed with ITS regions [29]. A recent study used chloroplast regions of *trn*H-*psb*A spacer, *trn*K intron including *mat*K gene, *trn*L intron, *trn*L*-trn*F spacer, and *trn*Q-*rps*16 spacer to confirm the identity of plants cultivated under the name *Cinnamomum porrectum* in Munchen-Nymphenburg Botanical Garden [30]. Further, Yang and colleagues used PCR—Restriction fragment length polymorphism (PCR-RFLP) for rapid identification of the indigenous medicinal crop *C*. *osmophloeum* from an adulterant, *C*. *burmannii* [31]. The PCR-RFLP can be combined with the morphology-based method known as the deep convolutional neural network (CNN) developed for the same purpose [32].

While there are many advantages of using plant barcoding for species identification and resolving phylogenies, it has failed in the identification of some closely related species in some cases, for example *Curcuma* (Zingiberaceae) [33], *Calligonum* [34], Bromeliaceae [35], *Picea* [36], *Berberis* [37], and Lauraceae [38]. Therefore, the objective of this study was to assess the possibility of utilizing universally accepted DNA barcoding regions for molecular level identification of *Cinnamomum* species in Sri Lanka. Both intraspecific and interspecific diversity were assessed with universal barcoding regions, *rbc*L, *mat*K, and *trn*H*-psb*A.

## Materials and methods

### Sample collection

Permissions for sample collection were obtained from the Research Committee of the Department of Wildlife Conservation, Sri Lanka, and the Department of Forestry, Sri Lanka.

Leaf samples of seven wild *Cinnamomum* species were collected from the rainforests, the germplasm collections at the National Cinnamon Research and Training Center, Thihagoda, Palolpitiya and Dalpitiya Mid Country Research Station, Department of Export Agriculture (DEA) (S1 Table). Samples of the cultivated *C*. *verum* variety ‘Sri Gemunu’ were collected from a vegetative propagated plantation at Nillambe, Sub Research Station, DEA, and seed propagated materials at the National Cinnamon Research and Training Center, Thihagoda, Palolpitiya. The variety Sri Gemunu is one of the two *Cinnamomum* varieties released by the DEA, Sri Lanka. Three individual plants from each species were considered as biological replicates to assess the intraspecific genetic diversity and named as accessions 001, 002, and 003. Altogether, a total of twenty-four (24) samples were included for the study.

The identity of the collected samples was verified using typical morphological characters. Thirty mature leaves with no symptoms of pest or diseases were collected randomly from the first secondary branch of each studied tree, and leaf morphological characters were recorded following the cinnamon descriptors [20, 22]. Thirteen characters were recorded including four quantitative characters (leaf length, leaf width, petiole length, leaf weight—mean weight of ten mature leaves), and nine qualitative characters (leaf shape, apex, base, texture, margin, arrangement, and color, bark fragrant, and bark taste).

In addition to qualitative characters, the average leaf length, leaf width, leaf weight, and petiole length were calculated and recorded for proper identification purposes (S1 Table). The within-species diversity of the quantitative morphological traits were assessed using Minitab statistical software (18^th^ version). The one-way ANOVA procedure was used for the determination of the significant difference (*P*<0.05) of means for considered morphological characters.

The collected specimens were further verified with the voucher specimens at the National Herbarium, Sri Lanka. The standard herbarium specimens were prepared by mounting on herbarium sheets and deposited at the National Herbarium, Sri Lanka (S1 Table).

### DNA extraction and sequencing

Total genomic DNA from all twenty-four (24) leaf samples was extracted using the cetyltrimethylammonium bromide (CTAB) method [39] with minor modifications [40, 41]. Leaf samples were ground to a fine powder using liquid nitrogen and 200 mg of each sample was used for the extraction. After adding the lysis buffer, *C*. *sinharajaense* and *C*. *dubium* sample were incubated at 65 ^o^C while shaking for overnight for better lysis without which pipetting was not possible due to jelly like lysate, and the other samples were incubated for one hour while inverting the tubes every 10 minutes. The chloroform: isoamyl alcohol (24:1) extraction was repeated twice to improve the quality of extracted DNA. The extracted DNA samples were re-suspended in 30–50 μL nuclease-free water and stored at 4 ^o^C. DNA quality and quantity were assessed with NanoDrop (NanoDrop 2000 spectrophotometer, Thermo scientific) and running on 0.8% agarose gels with a voltage of 5 Vcm^-1^ for 30 minutes.

The polymerase chain reaction (PCR) was performed using standard universal plant DNA barcoding primers (Table 1) with a previously optimized reaction mixture [40, 42] containing lx PCR buffer, 1.5 mM MgCl_2_, 200 μM dNTP (Promega, USA), 0.2 μM of each primer (Integrated DNA Technologies, Singapore), 100 ng of DNA, 0.8 μM spermidine and 1 Unit Go Taq Flexi DNA polymerase (Promega, USA). The PCR cycle consisted of initial denaturation at 94°C for 2 minutes, followed by 35 cycles of 94°C for 1 minute, annealing at 50 ^o^C to 55°C for 30 seconds (Table 1) and 72°C for 30 seconds, and a final extension at 72°C for 3 minutes. Products were separated by electrophoresis (5 Vcm^-1^) on 1.5% agarose gels and stained with Ethidium Bromide. All the PCR products were sent to Macrogen Inc (Seoul, South Korea-http://dna.macrogen.com) for bi-directional Sanger sequencing using the same primers used for PCR.

**Table 1:** Primers used for DNA barcoding studies.

Region	Primer	Sequence 5’-3’	Annealing temperature	Reference
*rbc*L	rbcLaf-M13_F	5’ TGT AAA ACG ACG GCC AGT ATG TCA CCA CAA ACA GAG ACT AAA GC 3’	55	[43]
	rbcLar-M13_R	5’ CAG GAA ACA GCT ATG ACG TAA AAT CAA GTC CAC CRC G 3’	55	[44]
*Mat*K	MatK_F(390F)	5’ CGA TCT ATT CAT TCA ATA TTT C 3’	55	[45]
	MatK_R(1326R)	5’ TCT AGC CAC GAA AGT CGA AGT 3’	55	[45]
*trn*H- *psb*A	psbAF	GTTATGCATGAACGTAATGCTC	50	[46]
	trnHR	CGCGCATGGTGGATTCACAATC	50	[46]

#### Additional sequences used for the analysis

The *trn*H*-psb*A intergenic regions of the accession 001 of all the species were extracted from high-throughput DNA sequencing data stored in a local database. For that, cleaned Sanger sequence data of accession 002 and 003 of each species were used as a query sequence to search (Blastn) high throughput sequence reads of the first accession of respective species using Geneious Prime Software (2020). The same approach was used to assess the quality of Sanger sequencing data of *rbc*L and *mat*K regions of the accession 001 of all the species. In addition, published sequences of *rbc*L, *mat*K, and *trn*H*-psb*A of *C*. *verum* were extracted from the chloroplast sequences deposited in the GenBank (NC_035236.1).

### Molecular data analysis

PCR amplification success rates of each region were calculated following the previously described method by Kress [47]. Accordingly, the success rate of PCR amplification refers to the percentage of successful individuals (of the total 24) in two PCR attempts. Both forward and reverse sequencing chromatograms were visually inspected using Geneious Prime software for sequencing errors. The 5’ and 3’ noisy sequences of about 30 bp were removed and cleaned bi-directional sequences of 470 bp and 796 bp were obtained for *rbc*L and *mat*K regions respectively. However, the reverse sequencing reaction continuously failed in *trn*H*-psb*A due to homopolymer runs after 320 bp. Therefore, even though the PCR product size was around 500 bp, the successful sequencing data were obtained only for 308 bp. The consensus sequences for all the samples have been submitted to GenBank (S2 Table).

The alignments were done separately for the three barcode regions, ends were trimed and then only three regions were joined including 470 bp, 796 bp, and 308 bp regions from *rbc*L, *mat*K, and *trn*H*-psb*A respectively. The same regions were included from *C*. *verum*, NC_035236.1. In each analysis, the multiple sequence alignment was carried out with Geneious Prime Software using Geneious alignment. The alignment was then exported to Molecular Evolutionary Genetics Analysis (MEGA-X) software for phylogenetic analysis. The maximum likelihood trees were constructed for *mat*K, and *trn*H*-psb*A data separately and the combined data set of *rbc*L, *mat*K, and *trn*H*-psb*A with the Tamura–Nei genetic distance model with 100 bootstrap replicates for node supports. A discrete Gamma distribution was used to model evolutionary rate differences among different sites [48, 49]. The intra and interspecific diversities were calculated for each region using the Tamura-Nei model of MEGA–X. All positions with less than 95% site coverage were eliminated and all three codon positions and the noncoding regions were included. Inter and intraspecific sequence divergences for combined barcode regions were also calculated using MEGA–X.

Further, Automatic Barcode Gap Discovery (ABGD) method described by Puillandre *et al* (2012) delaminate species based on the divergence among organisms [50]. For that, multiple sequence alignment files of *mat*k, and *trn*H-*psb*A were separately uploaded to AGBD web (https://bioinfo.mnhn.fr/abi/public/abgd/abgdweb.html). The JC69 Jukes-Cantor measure was set at prior minimum (Pmin) and prior maximum (Pmax) divergence of intraspecific diversity at 0.001 and 0.1 respectively.

## Results

We used the standard morphological traits for proper identification of *Cinnamomum* species and to confirm the identity of collected specimens. The leaf traits such as leaf shape, apex, base, and venation were different among *C*. *litseifolium*, *C*. *citriodorum*, *C*. *sinharajaense*, *C*. *ovalifolium*, and *C*. *capparu-coronde*. *C*. *sinharajaense* has a unique elliptic shape with a narrowly-acuminate apex. *Cinnamomum citriodorum* shows prominent pinnate venation pattern while all the other species have shown a three-vein structure (S1 Table). Of them, *C*. *ovalifolium*, and *C*. *litseifolium* have short basal veins while others have long basal veins reaching the leaf apex (S1 Table). The entire leaf margin is prominent in all species. The flat-leaf blade is also prominent in all species except *C*. *litseifolium* where it is slightly twisted. However, *C*. *rivulorum*, and *C*. *dubium* could not easily distinguish each other using leaf morphological characters. Line diagrams of leaf shape, apex, base, and venation of each accession are given in S1 Table. As such, a considerable intraspecific diversity was reported in leaf shape, leaf apex, and leaf base in all the species. The quantitative traits such as leaf length, leaf width, petiole length, and leaf weight also varied considerably within and among species. Nevertheless, the intraspecific diversity was significant only in *C*. *sinharajaense*, *C*. *dubium*, and *C*. *citriodorum* (S1 Table). For example, *C*. *sinharajaense* grown in Sinharaja rainforest had significantly longer leaf petioles and longer, wider and heavier leaves compared to the same species grown in Matara (18 cm). However, all three accessions had typical qualitative characteristics to be considered as *C*. *sinharajaense*. *Cinnamomum dubium* collected from Sinharaja rainforest had significantly longer leaf petioles and leaves compared to the accession collected from Matara.

Leaf morphology of the collected samples were further confirmed with herbarium specimens at the National Herbarium. All specimens used in the current study had typical morphological characteristics described previously for species identification purposes.

Good quality DNA could be extracted from all the samples with the modified DNA extraction protocol [40, 41]. PCR success is an important factor considered in selecting barcoding regions [23]. The PCR success rates calculated as the percentage of samples resulting expected band size in two attempts were 100% for *rbcL* and *matK* regions and 75% for *trn*H*-psb*A region (Table 2). All the samples resulted good quality forward and reverse sequencing data for *rbc*L and *mat*K regions. However, the reverse reaction of all *trn*H-*psb*A sequencing attempts were failed after 320 bp due to homopolymer stretch of T, and therefore, only 308 bp with successful forward and reverse regions were included in the analysis. The alignments done for each region identified polymorphic sites (Table 2). The variability in the *rbc*L was limited to only one site, at the 56^th^ position, found only in *C*. *ovalifolium* accession 001. The *trn*H-*psb*A region consists of the highest number of variable sites (11) while *mat*K region had four variable sites. Altogether, 16 variable sites were included in the analysis (Tables 2 and 3). The intraspecific and interspecific diversity indices were calculated for each region (Table 2). Only for *rbc*L, intraspecific diversity was higher than interspecific diversity.

**Table 2:** Molecular features of *Cinnamomum* species barcode.

Region	PCR amplification success rate %	^a^Average sequence length (bp)	^b^Sequence alignment length (bp)	No. of variable sites %	Interspecific diversity %	Intraspecific diversity %
**rbcL**	**100**	^**470**^	^**470**^	**0.21**	**7.7E-06**	**0.00018**
					**±0.000007**	**±0.000174**
**MatK**	**100**	^**796**^	^**796**^	**0.5**	**0.00093**	**0.00053**
					**±0.000740**	**±0.000320**
**trnH psbA**	**75**	^**308**^	^**308**^	**3.57**	**0.011709±**	**0.00034**
					**0.0032**	**±0.001100**

^a^ length of sequencing data after trimming the noisy 5’ and 3’ ends

^b^ length after multiple alignments.

**Table 3:** Sequence variation of *mat*K, *rbc*L, and *trn*H-*psb*A regions of *Cinnamomum* species.

*Region*	*rbc*L	*Mat*K				*trn*H *psb*A										
*Position*	56	52	353	357	795	30	82	83	85	86	89	118	149	154	181	380
*C*. *cap* 001	C	T	T	T	C	T	A	A	A	G	A	C	C	T	C	T
*C*. *cap* 002	C	C	C	T	C	T	A	A	A	G	A	C	A	A	A	C
*C*. *cap* 003	C	T	T	T	C	T	A	A	A	G	A	C	C	T	C	T
*C*. *cit* 001	C	T	C	T	C	T	A	A	A	G	A	C	C	A	C	T
*C*. *cit* 002	C	T	C	T	C	T	A	A	A	G	A	C	C	A	C	T
*C*. *cit* 003	C	T	C	T	C	T	A	A	A	G	A	C	C	A	C	T
*C*. *dub* 001	C	C	C	T	T	T	G	T	C	T	T	T	A	A	A	C
*C*. *dub* 002	C	C	C	T	T	T	A	A	A	G	A	C	A	A	A	C
*C*. *dub* 003	C	C	C	T	T	T	G	T	C	T	T	T	A	A	A	C
*C*. *lit* 001	C	T	C	T	C	T	A	A	A	G	A	C	C	A	C	T
*C*. *lit* 002	C	C	C	T	C	T	A	A	A	G	A	C	C	A	C	T
*C*. *lit* 003	C	T	C	G	C	T	A	A	A	G	A	C	C	A	C	T
*C*. *ova* 001	T	T	C	T	C	T	A	A	A	G	A	C	C	A	C	T
*C*. *ova* 002	C	T	C	T	C	T	A	A	A	G	A	C	C	A	C	T
*C*. *ova* 003	C	T	C	T	C	T	A	A	A	G	A	C	C	A	C	T
*C*. *riv* 001	C	C	C	T	T	G	A	A	A	G	A	C	A	A	A	C
*C*. *riv* 002	C	C	C	T	T	G	A	A	A	G	A	C	A	A	A	C
*C*. *riv* 003	C	C	C	T	T	G	A	A	A	G	A	C	A	A	A	C
*C*. *sin* 001	C	T	C	T	C	T	G	T	C	T	T	T	C	A	C	T
*C*. *sin* 002	C	C	C	T	C	T	G	T	C	T	T	T	A	A	A	C
*C*. *sin* 003	C	C	C	T	C	T	G	T	C	T	T	T	A	A	A	C
*C*. *ver* 001	C	C	C	T	C	T	G	T	C	T	T	T	A	A	A	C
*C*. *ver* 002	C	C	C	T	C	T	G	T	C	T	T	T	A	A	A	C
*C*. *ver* 003	C	C	C	T	C	T	G	T	C	T	T	T	A	A	A	C
*C*. *ver* (NCBI)	C	C	C	T	C	T	G	T	C	T	T	T	A	A	A	C

*C*. *cap* 001, *C*. *capparu-coronde* 001- KGG.BS-2018-8-CC-M-1; *C*. *cap* 002, *C*. *capparu-coronde* 002- KGG.BS-2018-8-CC-M-2; *C*. *cap* 003, *C*. *capparu-coronde* 003- RAAK.BS-2018-9-CC-D-1; *C*. *cit* 001, *C*. *citriodorum* 001*-* KGG.BS-2018-8-C-M-1; *C*. *cit* 002, *C*. *citriodorum* 002*-* BS-2019-5-C-N-1; *C*. *cit* 003, *C*. *citriodorum* 003*-* BS-2019-5-C-N-2; *C*. *dub* 001, *C*. *dubium* 001*-*RHG.BS-2018-11-D-S-1; *C*. *dub* 002, *C*. *dubium* 002*-* KGG.BS-2018-8-D-M-1; *C*. *dub* 003, *C*. *dubium* 003*-* RHG.BS-2018-11-D-S-2; *C*. *lit* 001, *C*. *litseifolium* 001- DSA.PCG.BS-2018-5-L-H-1; *C*. *lit* 002, *C*. *litseifolium* 002- KGG.BS-2018-8-L-M-1; *C*. *lit* 003, *C*. *litseifolium* 003- RAAK.BS-2018-9-L-D-1; *C*. *ova* 001, *C*. *ovalifolium* 001*-* DSA.PCG.BS-2018-5-O-H-1; *C*. *ova* 002, *C*. *ovalifolium* 002*-*DSA.PCG.BS-2018-5-O-HP-1; *C*. *ova* 003, *C*. *ovalifolium* 003*-*DSA.PCG.BS-2018-5-O-HP-1; *C*. *riv* 001, *C*. *rivulorum* 001*-* KGG.BS-2018-8-R-M-1; *C*. *riv* 002, *C*. *rivulorum* 002*-* KGG.BS-2018-8-R-M-2; *C*. *riv* 3, *C*. *rivulorum* 003*-* KGG.BS-2018-8-R-M-3; *C*. *sin* 1, *C*. *sinharajaense* 001*-* RHG.BS-2018-11-S-S-1; *C*. *sin* 002, *C*. *sinharajaense* 002*-* KGG.BS-2018-8-S-M-1; *C*. *sin* 003, *C*. *sinharajaense* 003*-* KGG.BS-2018-8-S-M-4; *C*. *ver* 001, *C*. *verum* 001*-* NL.BS.CHWMRB-2018-6-V-N; *C*. *ver* 2, *C*. *ver* 002*-* KGG.BS-2018-8-V-M-1; *C*. *ver* 003, *C*. *ver* 003*-* KGG.BS-2018-8-V-M-2; *C*. *ver*, *C*. *verum* (NC_035236.1).

Interestingly, both *C*. *dubium* and *C*. *rivulorum* have identical *mat*K regions, with no interspecific variability among accessions. Similarly, both *C*. *verum and* two accessions of *C*. *sinharajaense* shared identical *mat*K regions. The intraspecific variation in *mat*K region was only observed in *C*. *capparu-coronde* and *C*. *litseifolium* while *C*. *litseifolium* had unique sequences for each accession (Table 3). *C*. *litseifolium*, *C*. *ovalifolium* and *C*. *citriodorum* had identical *trn*H-*psb*A regions with no interspecific variability (Table 3). Interestingly, *C*. *verum* and *C*. *sinharajaense* had identical *trn*H-*psb*A sequences except in one *C*. *sinharajaense* accession, collected from Sinharaja rainforest. Interestingly, one *C*. *capparu-coronde* accession and one *C*. *dubium* accession shared identical *trn*H-*psb*A regions.

**Fig 1:**
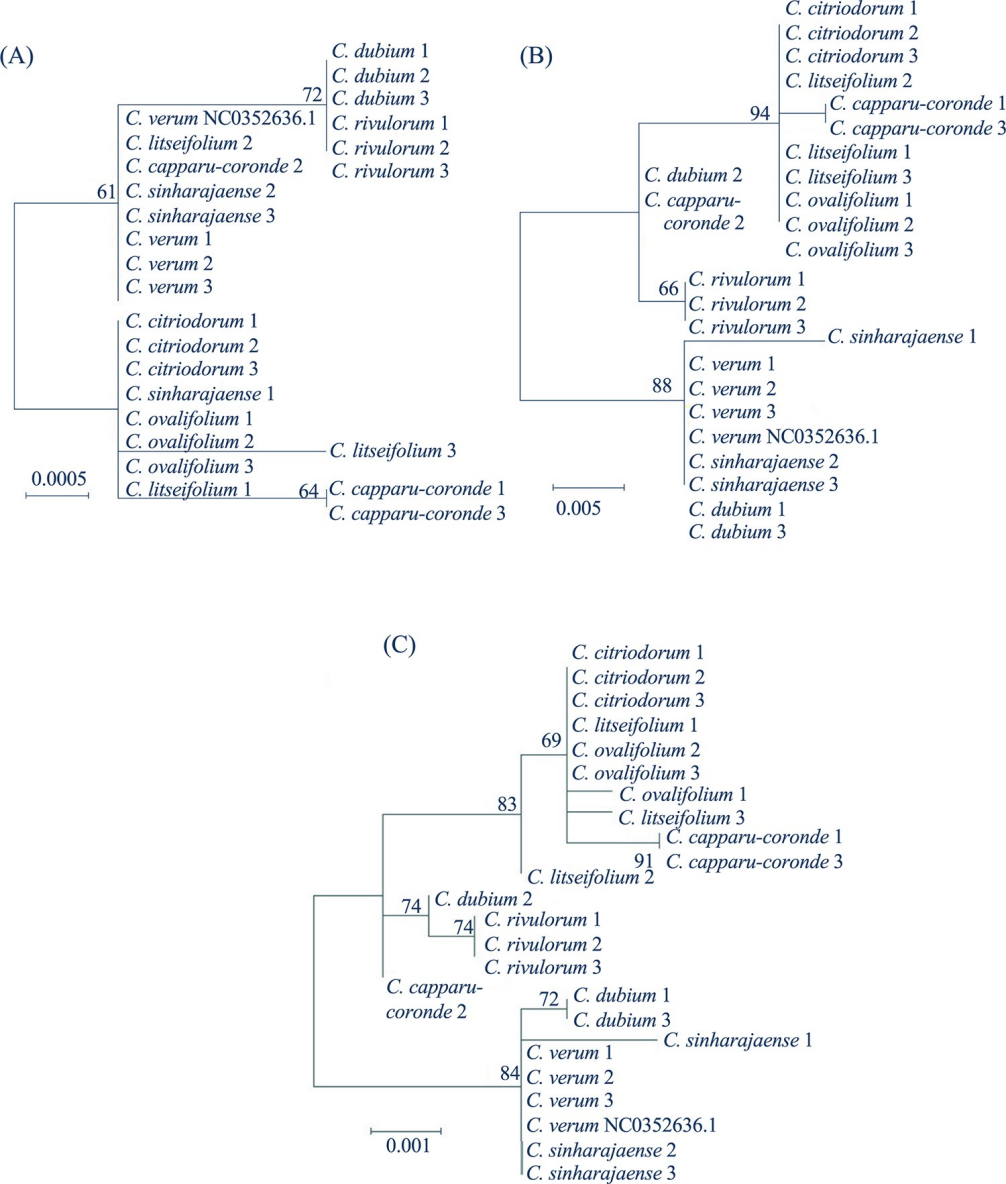
Maximum likelihood trees (unrooted) inferred from DNA barcode data for *Cinnamomum* species. (A) matk (B) trnH-psbA (C) combined data sets of rbcL, matK, and trnH-psbA regions. Values on branches represent bootstrap values.

The same relationship was reflected in the unrooted maximum-likelihood trees constructed with complete alignments of *mat*k (Fig 1(A)), *trn*H-*psb*A (Fig 1(B)) and all three regions (Fig 1(C)). The phylogeny of *Cinnamonum* species could not be resolved either with a single sequence barcode of either *mat*K or *trn*H-*psb*A or combined barcodes of all three regions. *Cinnamomum verum* and *C*. *sinharajaense* grouped in the combined analysis and the analysis done with *trn*H-*psb*A region. However, when the tree was built with *mat*K region, the *C*.*sinharajaense* accession (001) did not group with other *C*. *sinharajaense* accessions. *Cinnamomum dubium* accessions grouped only in the tree built with *mat*K region while the accession 002 grouped separately in the phylogenetic trees drawn from *trn*H-*psb*A and combined sequences. In the combined analysis, all three accessions of *C*. *citriodorum*, *C*. *rivulorum*, and *C*. *verum* grouped together.

Intraspecific sequence divergence was calculated from the combined sequences of each species. It is calculated as the number of base substitutions per site in averaging all the sequence pairs. This analysis also confirmed no within-species sequence divergence in *C*. *citriodorum*, *C*. *rivulorum*, and *C*. *verum*. The highest intraspecific sequence divergence was observed in *C*. *capparu-coronde* and *C*. *dubium* (Table 4). The interspecific evolutionary divergence was estimated considering all three accessions from each species as a group (Table 5). The number of base substitutions per site calculated by averaging over all sequence pairs between groups show the highest estimated sequence divergence between *C*. *ovalifolium* and the cultivated species *C*.*verum*. The lowest sequence divergence was observed between *C*. *sinharajaense* and *C*.*verum*.

**Table 4:** Intraspecific sequence divergence.

*Cinnamomum* species	Sequence divergence ± Standard Error
*C*. *verum*	0
*C*. *sinharajaense*	0.00126±0.0007195
*C*. *rivulorum*	0
*C*. *ovalifolium*	0.00042±0.0003388
*C*. *litseifolium*	0.00084±0.0006215
*C*. *dubium*	0.00253±0.0011095
*C*. *citriodorum*.	0
*C*. *capparu-coronde*	0.00253±0.0009701

**Table 5:** Interspecific sequence divergence between species.

*Cinnamomum* species	*C*. *verum*	*C*. *sinharajaense*	*C*. *rivulorum*	*C*. *ovalifolium*	*C*. *litseifolium*	*C*. *dubium*	*C*. *citriodorum*.	*C*. *cappru-coronde*
*C*. *verum*		0.0004	0.0013	0.0018	0.0017	0.0007	0.0018	0.0016
*C*. *sinharajaense*	0.0006		0.0014	0.0016	0.0015	0.0008	0.0016	0.0016
*C*. *rivulorum*	0.0051	0.0057		0.0016	0.0016	0.0010	0.0016	0.0014
*C*. *ovalifolium*	0.0066	0.0059	0.0040		0.0003	0.0017	0.0002	0.0009
*C*. *litseifolium*	0.0063	0.0057	0.0038	0.0006		0.0016	0.0003	0.0009
*C*. *dubium*	0.0019	0.0025	0.0032	0.0059	0.0057		0.0016	0.0014
*C*. *citriodorum*	0.0063	0.0057	0.0038	0.0002	0.0004	0.0057		0.0008
*C*. *capparu-coronde*	0.0063	0.0061	0.0038	0.0019	0.0020	0.0057	0.0017	

**Fig 2:**
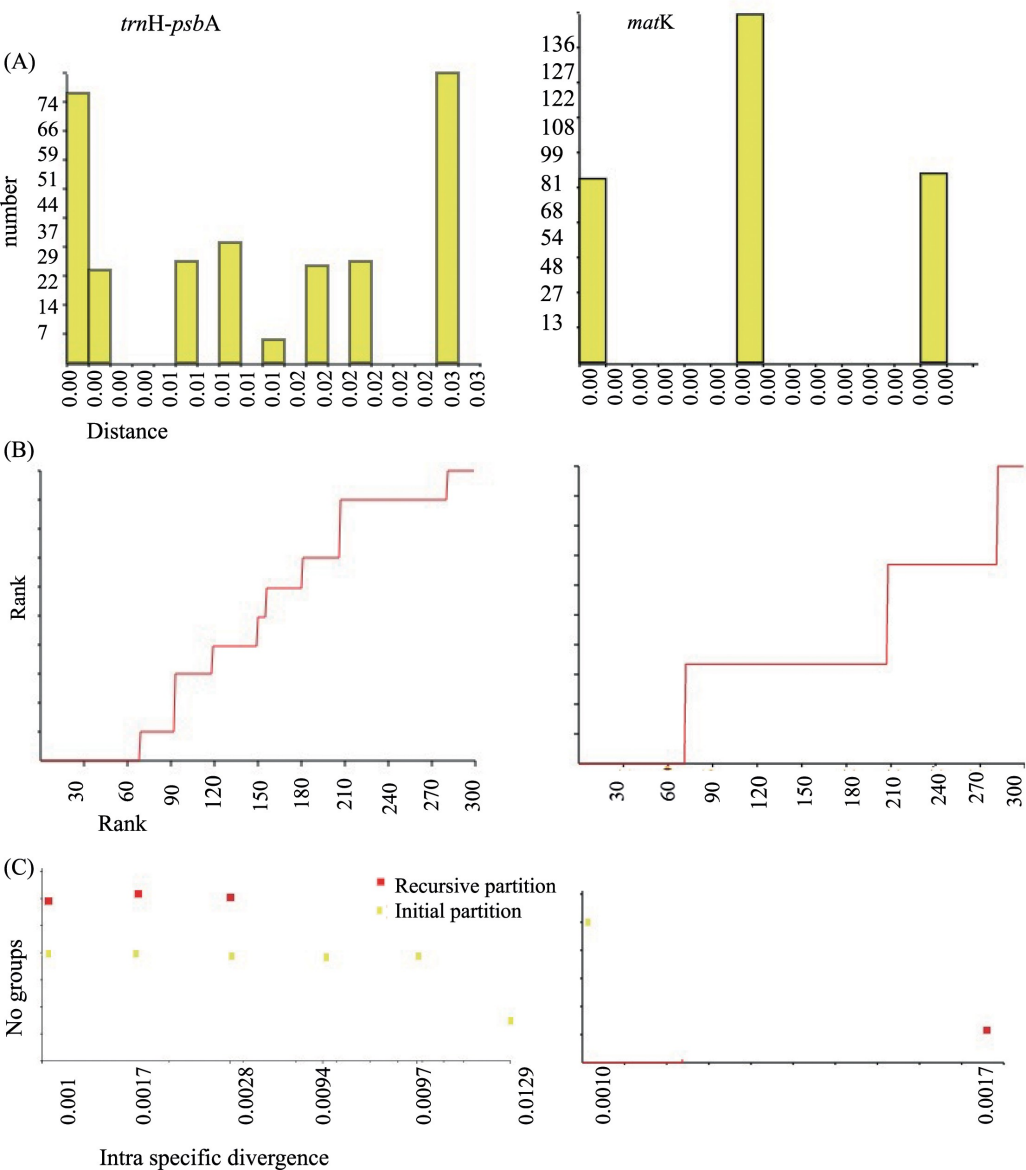
Schematic illustration of Automatic Barcode Gap Discovery (ABGD) with *mat*K and *trn*H-*psb*A regions for *Cinnamomum* species. (A) Distribution of pairwise differences. Low divergence represent intraspecific divergence, whereas higher divergence represents interspecific divergence (B) Ranked pairwise differences (C) Grouping.

The automatic barcode gap discovery method could not identify clear barcode gap either in *mat*k or in *trn*H-*psb*A region. The number of groups ranges from 1 to 6 with *trn*H-*psb*A while it ranges from 1 to 5 with *mat*k (Fig 2).

## Discussion

All the species included in the study are endemic to Sri Lanka. While the other seven are still in the wild, *C*. *verum* has been cultivated since 1500 AC. So far, the species identification and nomenclature have completely been done with morphological traits, highly affected by the environment. Our results also showed the same, for example having significantly different leaves when *C*. *sinharajaense* is grown in the Sinharaja rainforest compared to the same grown in the germplasm collection at Matara. Therefore, we wanted to confirm the identity of the collected samples before molecular analysis. We used standard leaf traits as much as possible [22] and further confirmed with the National Herbarium, Sri Lanka. For example, eventhough *C*. *sinharajaense* collected from Sinharaja rainforest had significantly larger leaves with longer petiols, qualitative traits, such as leaf apex, base, shape and venation match with typical leaf morphology of the species [22].

DNA barcoding has been used widely in plant biology to resolve phylogenies of related taxa. Nucleotide variation in these standard regions was sufficient to distinguish even closely related taxa. However, in some cases, it was reported that the nucleotide variability in the standard regions was not sufficient to distinguish closely related taxa [33]. It is known that the variability in the *rbc*L region is relatively lower compared to the *mat*K and *trn*H-*psb*A regions [51]. Our data also agree with the claim, having only one variable site in the *rbc*L region among the local species. It was found only in one of the collected accessions of *C*. *ovalifolium*. However it is a synonymous substitution. Since the variable site was present only in one accession, the intraspecific diversity of *C*.*ovalifolium* was higher than that of interspecific.

DNA barcoding has been suggested as the method of choice for species differentiation purposes of *Cinnamomum*. For example, Swetha *et al* (2014) suggested utilizing the barcoding loci *rbc*L, *mat*K, and *psb*A-*trn*H to detect the presence of adulterants such as *C*. *aromaticum* and *C*. *malabathrum* (Lam.) J. Presl in traded samples of cinnamon [52].

Nevertheless, our data suggest that the variation in standard barcoding regions *rbc*L, *mat*K, and *trn*H*-psb*A individually or in combination are not sufficient to discriminate *Cinnamomum* species found in Sri Lanka. While there is only a single base substitution in one *C*. *ovalifolium* accession, the other two accessions of the same species and all *C*. *citriodorum* accessions consist of identical barcode regions. However, those two species have distinct morphological features such as pinnate venation pattern with no issue in distinguishing at the field level [22]. Both high-performance liquid chromatography (HPLC) and Gas chromatography-mass spectrometry (GC-MS) analysis currently underway show that they also have distinct chemical profiles.

Except for one accession of *C*. *sinharajaense* collected from Sinharaja rainforest, the other two accessions of the same species, and all four *C*. *verum* accessions included in the analysis had identical barcoding regions considered. *Cinnamomum sinharajaense* can easily be distinguished from others using leaf traits such as large elliptic leaves and reticulated secondary venation which is visible on both sides of the blade [22]. Prevoius work also suggest such intraspecific morphological diversity depends on the environment [14]. Leaves are larger with more acuminate apex when the plants are grown under shaddy conditions. While *C*. *sinharajaense* accession 001 collected from Sinharaja rain forest grown under high shade conditions, accession 002 and 003 were collected from Matara grown under open environment. Germplasm collection in Matara has been established about 10 years ago from seeds collected from Sinharaja forest. Probably both accession 002 and 003 were originated from seeds collected from single mother plant. Our recent work showed that *C*. *zeylanicum* offspring resulted from a single cross pollination event are genetically and morphologically divese [53]. Nevertheless, HPLC and GC-MS analysis suggest all *C*. *sinharajaense* accessions and *C*. *verum* accessions consist of very similar chemical profiles except the differences in concentrations. Such concentration differences are very common in *C*. *zeylanicum* [54].

Results suggested high intraspecific diversity in some species. For example, each accession of C. *litseifolium* has unique *mat*K sequences, and one of them was similar to the *mat*K region of *C*. *ovalifolium*. Further, the nucleotide substitution at 119^th^ position resulted change in amino acid from tryptophan to glycine (S1 Fig). However, no intraspecific diversity among three accessions collected from *C*. *citriodorum*, *C*. *rivulorum* and *C*. *verum*. The *Cinnamomum* flower is naturally adapted for cross-pollination with protogynous dichogamy behavior [2, 55]. The same flower is opened twice, the first day, as a female flower with receptive stigma to be pollinated with mature pollen from another flower/s. The same flower will be opened the next day as a male flower with mature pollen. However, our recent work showed that, overlapping period of 45 min to 1 hr for the two flower types of the same tree [56]. Therefore, self-pollination with pollen from the same plant is also a possibility.

The lowest sequence divergence was recorded between cultivated species, *C*. *verum* and *C*. *sinharajaense*. Though they are morphologically distinct, they share similar chemical profiles. *Cinnamomum sinharajaense* is naturally grown only in the Sihnaraja rainforest with limited populations. The highest interspecific divergence was found between *C*. *ovalifolium* and *C*. *verum*. Interestingly, *C*. *ovalifolium* is only found in upcountry rainforests above 1000 m in the central highlands of Sri Lanka. The historical evidence suggests that the cultivated species, *C*. *verum* was also naturally grown in upcountry rainforests before domestication.

The barcode gap sorts the sequences into species whenever the divergence among organisms belonging to the same species is smaller than divergence among organisms from different species [50]. The barcode gap of *mat*K or *trn*H-*psb*A could not discriminate *Cinnamomum* species in Sri Lanka.

Overall, the discriminating power of standard barcoding regions selected, *rbc*L, *mat*K, *trn*H-*psb*A are not sufficient even to distinguish morphologically distinct *Cinnamomum* species found in Sri Lanka and therefore the identity of true cinnamon and its wild relatives couldn’t be assessed. Nevertheless, the analyses suggest genetic closeness among eight *Cinnamomum* species found in Sri Lanka. Some of the wild species such as *C*. *litseifolium* and *C*. *rivulorum* are categorized as endangered species in Sri Lanka while all other species are vulnerable [57]. Therefore, correct identification is essential for both conservation and sustainable utilization of valuable genetic resources. The identification of alternative chloroplast barcoding region/s would be important. The complete chloroplast genome sequencing alone or together with fast-evolving genomic regions such as internal transcribed spacer (ITS) regions may provide sufficient molecular data for identifying closely related *Cinnamomum* species. Complete chloroplast genome analysis could distinguish closely related species such as *Pterocarpus* [58] and *Fritillaria* [59]. Therefore, such an approach is proposed for distinguishing *Cinnamomum* species in Sri Lanka.

## Supporting information

S1 Table Details of specimens included in the study. Data presented as mean ± standard error of the mean of the three replicates. Mean values represented by different lower case letters within a species in a given column refer to significant differences (*P*<0.05). LL, leaf length (cm); LW, leaf width (cm); W, leaf weight (g); PL, petiole length (mm); size bar, 10 cm.
(PDF)

S2 Table NCBI accession numbers of *Cinnamomum* species studied. (PDF)

S1 Fig Amino acid sequence alignment of *mat*K region for *Cinnamomum* species. (PDF)
