# Supplementary material for: Universal barcoding regions, rbcL, matK and trnH-psbA do not discriminate Cinnamomum species in Sri Lanka
Source: PLoS One. 2021 Feb 10;16(2):e0245592. doi: 10.1371/journal.pone.0245592 (PMC7875411; doi:10.1371/journal.pone.0245592)
Supplement: S1 Table — Data presented as mean ± standard error of the mean of the three replicates. Mean values represented by different lower case letters within a species in a given column refer to significant differences (P<0.05). LL, leaf length (cm); LW, leaf width (cm); W, leaf weight (g); PL, petiole length (mm); size bar, 10 cm. (PDF) [file pone.0245592.s001.pdf]

Species	District	Location	Voucher. No	Qualitative leaf characters		Qualitative characters			
				Leaf shape, apex and base	Venation	LL	LW	PL	W
C. capparu-coronde 001	Matara	E-6.0263 N-80.5616	KGG.BS-2018-8-CC-M-1	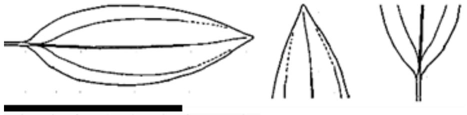	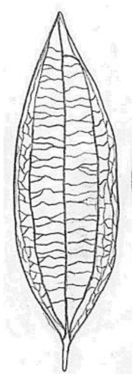	14.3 ±1.3^{a}	5.7± 0.9^{a}	1.7± 0.2^{a}	10.6 ±1.6^{a}
C. capparu-coronde 002	Matara	E-6.0249 N-80.5616	KGG.BS-2018-8-CC-M-2	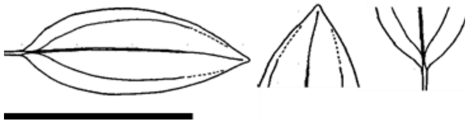		15.8 ±1.9^{a}	6.3± 0.8^{a}	1.9± 0.2^{a}	11.5 ±1.9^{a}
C. capparu-coronde 003	Kandy	E-7.1346 N-80.5851	RAAK.BS-2018-9-CC-D-1	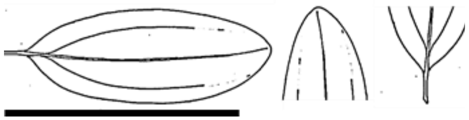		15.5 ±1.8^{a}	6.0± 0.7^{a}	1.8± 0.2^{a}	10.2 ±1.6^{a}
C. citriodorum 001	Matara	E-6.0225 N-80.5623	KGG.BS-2018-8-C-M-1	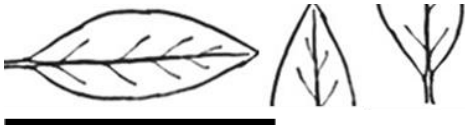	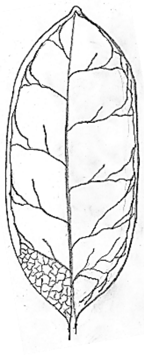	10.5 ±0.8^{a}	3.7± 0.3^{a}	1.7± 0.2^{a}	4.8± 1.5^{a}
C. citriodorum 002	N'eliya	E-6.7548 N-80.7204	BS-2019-5-C-N-1	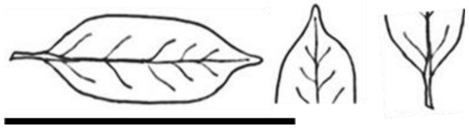		9.1± 1.9^{a}	3.6± 0.6^{a}	1.3± 0.2^{b}	4.6± 1.3^{a}
C. citriodorum 003	N'eliya	E-6.5431 N-80.4344	BS-2019-5-C-N-2	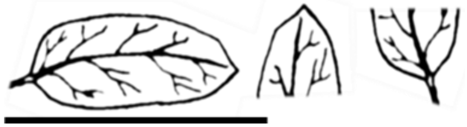		8.9± 1.5^{a}	3.4± 0.5^{a}	1.4± 0.2^{b}	4.3± 0.7^{a}

C. dubium 001	Rathnapur a	E-6.4108 N-80.5088	RHG.BS-2018-11-D-S-1	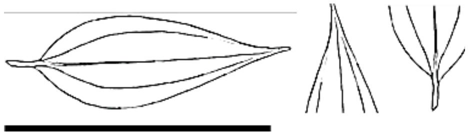	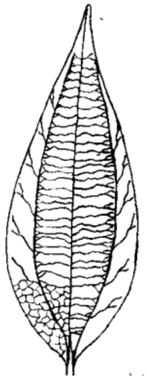	14.5 ±0.5[a]	4.1± 0.3[a]	1.6± 0.1[a]	6.9± 1.3[a]
C. dubium 002	Matara	E-6.0285 N-80.5610	KGG.BS-2018-8-D-M-1	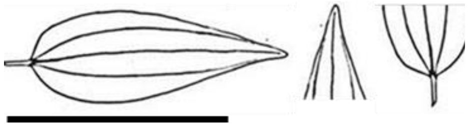		12.4 ± 1.2[b]	3.4± 0.5[a]	1.2± 0.2[b]	6.8± 0.4[a]
*C. dubium 00*3	Rathnapur a	E-6.4267 N-80.4146	RHG.BS-2018-11-D-S-2	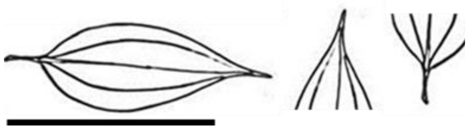		14.5 ±1.9[a]	3.9± 0.2[a]	1.6± 0.1[a]	7.2± 1.2[a]
C. litseifolium 001	N'eliya	E-6.9683 N-80.7700	DSA.PCG.BS-2018-5-L-H-1	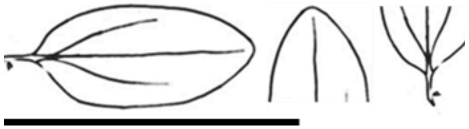	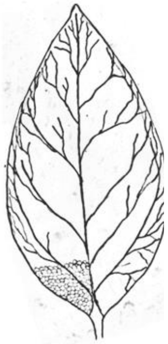	8.1± 0.5[a]	3.8± 0.3[a]	1.3± 0.1[a]	5.42 ±1.0[a]
C. litseifolium 002	Matara	E-6.0297 N-80.5600	KGG.BS-2018-8-L-M-1	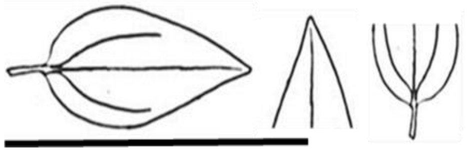		8.7± 0.3[a]	3.8± 0.3[a]	1.3± 0.2[a]	5.8± 1.8[a]
C. litseifolium 003	Kandy	E-7.1325 N-80.5879	RAAK.BS-2018-9-L-D-1	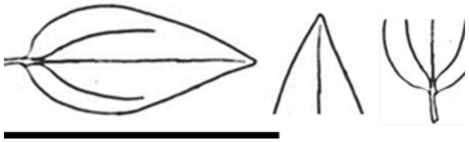		9.1± 0.7[a]	3.9± 0.4[a]	1.3± 0.2[a]	5.5± 1.9[a]

C. ovalifolium 001	N'eliya	E-6.9696 N-80.7700	DSA.PCG.BS-2018-5-O-H-1	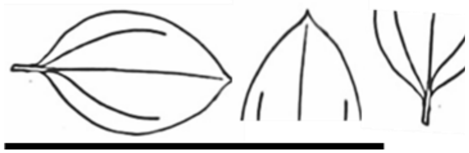	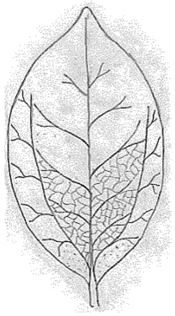	5.1 ± 0.9^{a}	3.3 ± 0.2^{a}	1.0 ± 0.2^{a}	3.4 ± 0.9^{a}
C. ovalifolium 002	N'eliya	E-6.8035 N-80.8031	DSA.PCG.BS-2018-5-O-HP-1	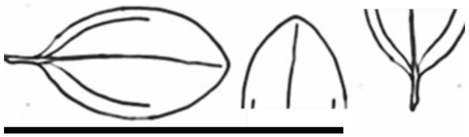		5.6 ± 0.6^{a}	3.4 ± 0.2^{a}	0.9 ± 0.1^{a}	3.5 ± 0.7^{a}
C. ovalifolium 003	N'eliya	E-6.8035 N-80.8031	DSA.PCG.BS-2018-5-O-HP-2	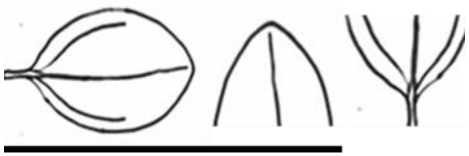		5.7 ± 0.3^{a}	3.4 ± 0.3^{a}	$1.\pm 0.1^{a}$	3.6 ± 0.6^{a}
C. rivulorum 001	Matara	E-6.0222 N-80.5624	KGG.BS-2018-8-R-M-1	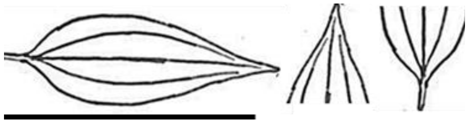	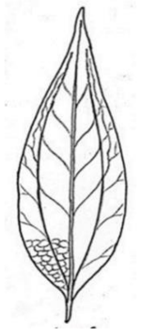	11.5 ± 1.3^{a}	4.3 ± 0.5^{a}	1.5 ± 0.1^{a}	5.2 ± 1.5^{a}
C. rivulorum 002	Matara	E-6.0222 N-80.5624	KGG.BS-2018-8-R-M-2	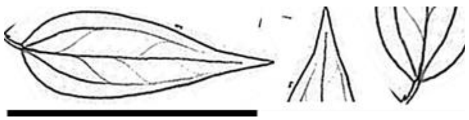		11.2 ± 0.7^{a}	4.3 ± 0.5^{a}	1.4 ± 0.2^{a}	5.1 ± 1.2^{a}
C. rivulorum 003	Matara	E-6.0222 N-80.5624	KGG.BS-2018-8-R-M-3	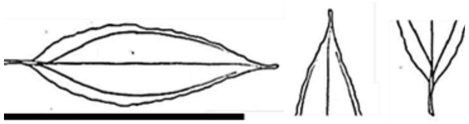		11.6 ± 0.8^{a}	4.3 ± 0.4^{a}	1.5 ± 0.1^{a}	4.5 ± 1.2^{a}

C. sinharajaense 001	Rathnapura	E-6.4352 N-80.4197	RHG.BS-2018-11-S-S-1	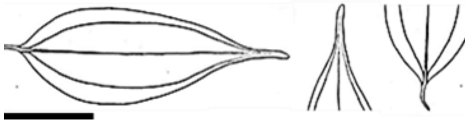	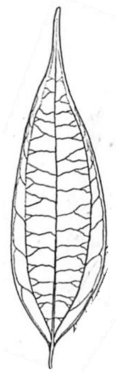	37.6 ±3.5^{a}	12.7 ±2.5^{a}	3.4± 0.5^{a}	36.4 ±4.2^{a}
C. sinharajaense 002	Matara	E-6.0239 N-80.5619	KGG.BS-2018-8-S-M-1	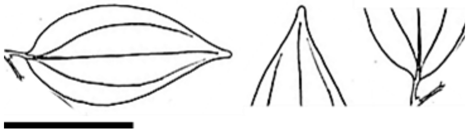		18.6 ±1.8^{b}	8.5± 1.3^{b}	2.2± 0.2^{b}	18.8 ±1.7^{b}
C. sinharajaense 003	Matara	E-6.0231 N-80.5619	KGG.BS-2018-8-S-M-4	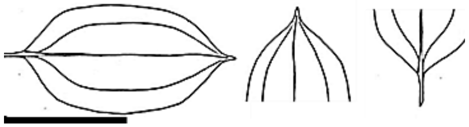		18.4 ±1.2^{b}	8.2± 1.1^{b}	2.2± 0.3^{b}	18.7 ±1.7^{b}
C. verum 001	Kandy	E-7.1991 N-80.5274	NL.BS. CHWMRB-2018-6-V-N-1	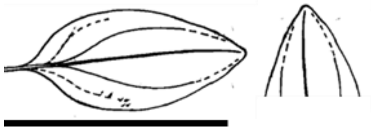	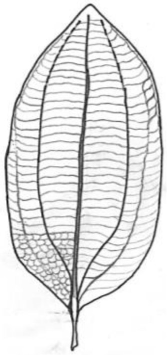	11.3 ±1.1^{a}	5.1± 0.6^{a}	1.5± 0.2^{a}	10.9 ±1.2^{a}
C. verum 002	Matara	E-6.0241 N-80.5654	KGG.BS-2018-8-V-M-1	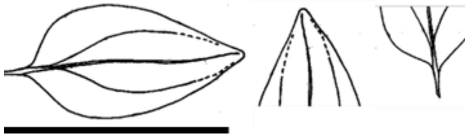		11.7 ±1.0^{a}	5.2± 0.5^{a}	1.5± 0.2^{a}	10.8 ±1.5^{a}
C. verum 003	Matara	E-6.0231 N-80.5624	KGG.BS-2018-8-V-M-2	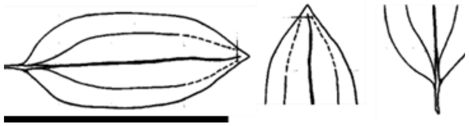		11.0 ±0.7^{a}	5.1± 0.3^{a}	1.5± 0.2^{a}	10.8 ±1.4^{a}
