# Supplementary material for: Universal barcoding regions, rbcL, matK and trnH-psbA do not discriminate Cinnamomum species in Sri Lanka
Source: PLoS One. 2021 Feb 10;16(2):e0245592. doi: 10.1371/journal.pone.0245592 (PMC7875411; doi:10.1371/journal.pone.0245592)
Supplement: S2 Table — (PDF) [file pone.0245592.s002.pdf]

Species/Specimen voucher	**Accession no**		
	ribulose-1,5-bisphosphate carboxylase/oxygenase large subunit (*rbc*L) gene, partial CDS, Chloroplast	maturase K (*mat*k) gene, partial cds; chloroplast	photosystem II protein D1 (*psb*A) gene, partial cds; *psb*A-*trn*H intergenic spacer, partial sequence; chloroplast
C. capparu-coronde 001 [Specimen_voucher=KGG.BS-2018-8-CC-M-1]	MW408292	MW408284	MW408322
C. capparu-coronde 002 [Specimen_voucher=KGG.BS-2018-8-CC-M-2]	MW408294	MW408285	MW408323
C. capparu-coronde 003 [Specimen_voucher=RAAK.BS-2018-9-CC-D-1]	MW408295	MW408286	MW408324
C. citriodorum 001 [Specimen_voucher=KGG.BS-2018-8-C-M-1]	MW408289	MW408281	MW408301
C. citriodorum 002 [Specimen_voucher=BS-2019-5-C-N-1]	MW408290	MW408282	MW408302
C. citriodorum 003 [Specimen_voucher=BS-2019-5-C-N-2]	MW408291	MW408283	MW408303
C. dubium 001 [Specimen_voucher=RHG.BS-2018-11-D-S-1]	MW386969	MW408278	MW408304
C. dubium 002 [Specimen_voucher=KGG.BS-2018-8-D-M-1]	MW408287	MW408279	MW408305
C. dubium 003 [Specimen_voucher=RHG.BS-2018-11-D-S-2]	MW408288	MW408280	MW408306
C. litseifolium 001 [Specimen_voucher=DSA.PCG.BS-2018-5-L-H-1]	MW386966	MW408275	MW408307
C. litseifolium 002 [Specimen_voucher=KGG.BS-2018-8-L-M-1] Chloroplast	MW386967	MW408276	MW408308

C. litseifolium 003 [Specimen_voucher=RAAK.BS-2018-9-L-D-1]	MW386968	MW408277	MW408309
C. ovalifolium 001 [Specimen_voucher=DSA.PCG.BS-2018-5-O-H-1]	MW386963	MW408272	MW408310
C. ovalifolium 002 [Specimen_voucher=DSA.PCG.BS-2018-5-O-HP-1]	MW386964	MW408273	MW408311
C. ovalifolium 003 [Specimen_voucher=DSA.PCG.BS-2018-5-O-HP-2]	MW386965	MW408274	MW408312
C. rivulorum 001 [Specimen_voucher=KGG.BS-2018-8-R-M-1]	MW386960	MW408269	MW408313
C. rivulorum 002 [Specimen_voucher=KGG.BS-2018-8-R-M-2]	MW386961	MW408270	MW408314
C. rivulorum 003 [Specimen_voucher=KGG.BS-2018-8-R-M-3]	MW386962	MW408271	MW408315
C. sinharajaense 001 [Specimen_voucher=RHG.BS-2018-11-S-S-1]	MW386957	MW408266	MW408316
C. sinharajaense 002 [Specimen_voucher=KGG.BS-2018-8-S-M-1]	MW386958	MW408267	MW408317
C. sinharajaense 003 [Specimen_voucher=KGG.BS-2018-8-S-M-4]	MW386959	MW408268	MW408318
C. verum 001 [Specimen_voucher=NL.BS. CHWMRB-2018-6-V-N-1]	MW386954	MW408263	MW408319
C. verum 002 [Specimen_voucher=KGG.BS-2018-8-V-M-1]	MW386955	MW408264	MW408320
C. verum 003 [Specimen_voucher=KGG.BS-2018-8-V-M-2]	MW386956	MW408265	MW408321
