# Supplementary material for: Universal barcoding regions, rbcL, matK and trnH-psbA do not discriminate Cinnamomum species in Sri Lanka
Source: PLoS One. 2021 Feb 10;16(2):e0245592. doi: 10.1371/journal.pone.0245592 (PMC7875411; doi:10.1371/journal.pone.0245592)
Supplement: S1 Fig — (PDF) [file pone.0245592.s003.pdf]

	1									10					120										130
1. C. cappau-coronde 001	I	L	I	P	H	P	I	H	L	E	K	T	L	W	L	F	K	D	P	F	M	H	Y	V	R
2. C. capparu-coronde 002	I	L	I	P	H	P	I	H	L	E	K	T	L	W	L	F	K	D	P	F	M	H	Y	V	R
3. C. capparu-coronde 003	I	L	I	P	H	P	I	H	L	E	K	T	L	W	L	F	K	D	P	F	M	H	Y	V	R
4. C. citriodorum 001	I	L	I	P	H	P	I	H	L	E	K	T	L	W	L	F	K	D	P	F	M	H	Y	V	R
5. C. citriodorum 002	I	L	I	P	H	P	I	H	L	E	K	T	L	W	L	F	K	D	P	F	M	H	Y	V	R
6. C. citriodorum 003	I	L	I	P	H	P	I	H	L	E	K	T	L	W	L	F	K	D	P	F	M	H	Y	V	R
7. C. dubium 001	I	L	I	P	H	P	I	H	L	E	K	T	L	W	L	F	K	D	P	F	M	H	Y	V	R
8. C. dubium 002	I	L	I	P	H	P	I	H	L	E	K	T	L	W	L	F	K	D	P	F	M	H	Y	V	R
9. C. dubium 003	I	L	I	P	H	P	I	H	L	E	K	T	L	W	L	F	K	D	P	F	M	H	Y	V	R
10. C. litseifolium 001	I	L	I	P	H	P	I	H	L	E	K	T	L	W	L	F	K	D	P	F	M	H	Y	V	R
11. C. litseifolium 002	I	L	I	P	H	P	I	H	L	E	K	T	L	W	L	F	K	D	P	F	M	H	Y	V	R
12. C. litseifolium 003	I	L	I	P	H	P	I	H	L	E	K	T	L	G	L	F	K	D	P	F	M	H	Y	V	R
13. C. ovalifolium 001	I	L	I	P	H	P	I	H	L	E	K	T	L	W	L	F	K	D	P	F	M	H	Y	V	R
14. C. ovalifolium 002	I	L	I	P	H	P	I	H	L	E	K	T	L	W	L	F	K	D	P	F	M	H	Y	V	R
15. C. ovalifolium 003	I	L	I	P	H	P	I	H	L	E	K	T	L	W	L	F	K	D	P	F	M	H	Y	V	R
16. C. rivulorum 001	I	L	I	P	H	P	I	H	L	E	K	T	L	W	L	F	K	D	P	F	M	H	Y	V	R
17. C. rivulorum 002	I	L	I	P	H	P	I	H	L	E	K	T	L	W	L	F	K	D	P	F	M	H	Y	V	R
18. C. rivulorum 003	I	L	I	P	H	P	I	H	L	E	K	T	L	W	L	F	K	D	P	F	M	H	Y	V	R
19. C. sinjharajaense 001	I	L	I	P	H	P	I	H	L	E	K	T	L	W	L	F	K	D	P	F	M	H	Y	V	R
20. C. sinharajaense 002	I	L	I	P	H	P	I	H	L	E	K	T	L	W	L	F	K	D	P	F	M	H	Y	V	R
21. C. sinharajaense 003	I	L	I	P	H	P	I	H	L	E	K	T	L	W	L	F	K	D	P	F	M	H	Y	V	R
22. C. ver 001	I	L	I	P	H	P	I	H	L	E	K	T	L	W	L	F	K	D	P	F	M	H	Y	V	R
23. C. ver 002	I	L	I	P	H	P	I	H	L	E	K	T	L	W	L	F	K	D	P	F	M	H	Y	V	R
24. C. ver 003	I	L	I	P	H	P	I	H	L	E	K	T	L	W	L	F	K	D	P	F	M	H	Y	V	R
25. C. ver (NCBI)	I	L	I	P	H	P	I	H	L	E	K	T	L	W	L	F	K	D	P	F	M	H	Y	V	R
